# Diagnostic value of nanopore-based metagenomic third-generation sequencing in the diagnosis of Pneumocystis jirovecii infection in patients with lung cancer

**DOI:** 10.1099/jmm.0.002031

**Published:** 2025-07-03

**Authors:** Yuyan Luo, Wei Cheng, Lei Ma, Tiantian Wang, Weirong Shi

**Affiliations:** 1 Department of Medical Oncology, Affiliated Nantong Hospital of Shanghai University, Nantong, Jiangsu 226001, PR China; 2 Department of Respiratory Diseases, Tongzhou People’s Hospital, Nantong, Jiangsu 226300, PR China; 3 Department of Respiratory Diseases, Affiliated Nantong Hospital of Shanghai University, Nantong, Jiangsu 226001, PR China; 4 Department of Thoracic Surgery, Affiliated Nantong Hospital of Shanghai University, Nantong, Jiangsu 226001, PR China

**Keywords:** diagnosis, lung cancer, metagenomics, nanopore-based third-generation sequencing, *Pneumocystis jirovecii*

## Abstract

**Introduction.** *Pneumocystis jirovecii* pneumonia (PJP, formerly known as *Pneumocystis carinii* pneumonia), an opportunistic fungal infection caused by the fungus *P. jirovecii*, is a severe pulmonary infection that primarily affects immunocompromised patients, including those with lung cancer. Traditional diagnostic methods for PJP, such as Grocott–Gomori’s methenamine silver staining and real-time PCR, have limitations, including low positivity and high missed diagnosis rates.

**Gap Statement.** Despite the critical need for accurate and sensitive diagnostic tools for PJP, especially in immunocompromised populations, existing methods fall short in providing the necessary reliability and efficiency.

**Aim.** This study aims to evaluate the efficacy of nanopore-based metagenomic third-generation sequencing in diagnosing *P. jirovecii* infection in lung cancer patients, hypothesizing that this approach may offer superior sensitivity and specificity.

**Methodology.** A prospective observational study was conducted on 118 lung cancer patients with suspected pulmonary * P. jirovecii* infection at the Sixth Hospital of Nantong City, China, from January 2021 to December 2023. The identification of pathogens in bronchoalveolar lavage fluid samples was performed using both metagenomics and traditional tests.

**Results.** Metagenomics showed a significantly higher detection rate of *P. jirovecii* (33.0%) compared to methenamine silver staining (4.2%) and real-time PCR (30.5%). The sensitivity, specificity and accuracy of metagenomics detection were all 100%, which is markedly superior to traditional methods. Furthermore, metagenomics also identified mixed infections with other pathogens, such as *Cytomegalovirus* and Epstein–Barr virus.

**Conclusion.** Metagenomics technology demonstrates high sensitivity and specificity in diagnosing *P. jirovecii* infection, including mixed infections with other pathogens, in lung cancer patients. It provides a clear direction for clinical treatment and is a powerful tool for diagnosing PJP, contributing to improved diagnostic efficiency and accuracy, reducing misdiagnosis and missed diagnosis rates and improving clinical outcomes in these patients.

**Impact Statement**

Nanopore-based metagenomics has revolutionized the diagnosis of *Pneumocystis jirovecii* (*P. jirovecii*) infections in lung cancer patients. This innovative method has significantly increased the detection rate to 33.0%, which is higher than that of traditional methods such as methenamine silver staining and real-time PCR. Notably, the sensitivity, specificity and accuracy of metagenomics in detecting *P. jirovecii* infections have reached an impressive 100%.

Moreover, metagenomics has proven to be highly precise in identifying mixed infections, which enhances diagnostic efficiency and facilitates targeted clinical interventions. This advancement is especially significant for immunocompromised patients, where early and accurate diagnosis is crucial for improving outcomes and reducing mortality rates.

The study’s findings highlight metagenomics as a powerful diagnostic tool that has the potential to transform the clinical management of *P. jirovecii* pneumonia in lung cancer patients. By contributing to better patient care, this approach may also reshape the field of infectious disease diagnostics.

## Data Summary

The authors confirm that all supporting data, code and protocols have been provided within the article.

## Introduction

*Pneumocystis jirovecii* pneumonia (PJP) (previously known as *Pneumocystis carinii* pneumonia) is an opportunistic fungal infection caused by *P. jirovecii*. PJP typically presents with fever, cough and dyspnoea, potentially progressing to respiratory failure, with characteristic imaging features of diffuse interstitial lung disease bilaterally [1,3]. Historically, PJP is associated with a higher incidence in patients with lung cancer. However, over the past few decades, its prevalence has significantly increased in non-lung cancer populations, such as those with other malignancies and autoimmune diseases, and in organ transplant recipients, due to the widespread use of immunosuppressive agents [4]. Lung cancer has the highest incidence and mortality amongst cancers worldwide. Retrospective studies from France (conducted between 1990 and 2010) reported a PJP incidence rate of 2.6 per 100,000 person-years amongst patients with lung cancer. In fact, patients with lung cancer accounted for 2% of all PJP cases, and 10.7% of PJP cases occurred in patients with solid tumour [5,6]. Further, a Japanese retrospective study (conducted between 2006 and 2018) showed that 30% of PJP cases associated with solid tumour were in patients with lung cancer [7]. Furthermore, Lee *et al*. showed that the 3-month mortality rate for patients with lung cancer with concurrent PJP infections was very high (61.6%), suggesting a poor prognosis [8]. Despite recent improvements in diagnosis and treatment, PJP remains a serious concern [9]. Microscopic examination – of tissue specimens, bronchoalveolar lavage fluid (BALF) or induced sputum – for the presence of *P. jirovecii* cysts and trophozoites (the active, vegetative form of the pathogen) remains the gold standard for diagnosing *Pneumocystis* pneumonia. Common staining methods for direct detection utilize Giemsa, methenamine silver and toluidine blue O stains, which are widely accepted as the most reliable diagnostic stains for *Pneumocystis* pneumonia [10]. In the absence of definitive microbiological evidence, clinical suspicion often relies on elevated serum 1,3-*β*-d-glucan (BDG; >80 ng l^−1^) in G-test results and significantly raised lactate dehydrogenase levels (>600 U l^−1^) in patients with bilateral ground-glass opacities on chest imaging [11,12]. The culture of the *Pneumocystis* genus remains challenging despite the development of an axenic culture technique – because of protocol complexity and need for differentiated pseudostratified airway epithelial cell line – limiting its practical application in routine diagnostic laboratories [13]. Furthermore, traditional staining and microscopic examination have low sensitivity due to strict specimen (lung tissue being optimal) and technical expertise requirements, leading to frequent underdiagnosis. Recently, PCR technology has been used for *Pneumocystis* diagnosis. It offers improved sensitivity (38%–52% higher than staining methods); however, false positives can occur [14]. Metagenomic next-generation sequencing can detect *P. jirovecii* sequences in BALF and peripheral blood. This, combined with clinical presentation and radiographic findings, allows for PJP confirmation. However, only a few clinical cases have been diagnosed with this method [15]. Nanopore-based sequencing, a third-generation sequencing technology, offers shorter turnaround times (≤6 h) and longer read lengths (1,000 bp to 4 Mb long), enhancing assembly efficiency and accuracy, thus addressing the limitations of short-read platforms in genomic sequencing [16,18]. Although nanopore-based sequencing initially had lower base-calling accuracy compared to second-generation sequencing, recent advancements have improved the accuracy rates (≤99 %), making it a promising future sequencing technology [19].

Here, we aim to evaluate the diagnostic value of nanopore-based metagenomic third-generation sequencing technology in detecting *P. jirovecii* infections in patients with lung cancer. We also explore its potential as a novel diagnostic tool for PJP and other microbial diseases co-occurring with lung cancer. Metagenomics is expected to enhance the diagnostic and therapeutic management of lung cancer in patients with concurrent PJP infections, thereby reducing misdiagnosis and missed diagnosis rates, and consequently lowering mortality.

## Methods

### Study design and participants

In this prospective observational study, we recruited a total of 118 hospitalized patients diagnosed with lung cancer and suspected to have PJP infection. This study was conducted at Nantong Sixth People’s Hospital between January 2021 and December 2023. Subjects who received targeted antibiotic therapy within 3 days prior to BALF sampling were excluded to avoid potential suppression of pathogen load, which could interfere with diagnostic accuracy. Patient demographic data, including gender, age, symptoms and imaging examination results, were collected (Table 1). The average age of the participants was 42.59±19.21 years (99 males and 19 females). The radiological diagnostic criteria for *Pneumocystis* pneumonia included typical manifestations of symmetric ground-glass opacities around bilateral hilar regions, with reticular or nodular patterns forming patchy or geographic distributions, extending from the hilum towards the peripheral lung fields with a tendency to coalesce. Atypical presentations involved multiple pulmonary nodules, lung cysts, pleural effusion, pneumothorax, enlarged hilar and mediastinal lymph nodes, amongst others. The main clinical features were low-grade fever, dry cough, progressive dyspnoea and hypoxaemia. BALF samples were collected from the patients for subsequent testing [20]. This procedure involved instilling 1–2 ml of 2% lidocaine into the target lung segment (the diseased lobe segment or right middle lobe or left upper lingular segment) via a biopsy channel to achieve local anaesthesia. Upon accessing the targeted bronchial segment or subsegment with the bronchoscope tip, sterile saline at 37 °C was rapidly infused through the working channel in volumes of 20–50 ml per aliquot (total volume of 60–120 ml). Following the infusion of saline, BALF was aspirated using appropriate negative pressure (<100 mmHg) and collected in a sterile container.

**Table 1.** Demographic and clinical characteristics of participants

Variable	Value (*N*=118)
Age (years)	42.59±19.21
Male:female	99 : 19
Fever	89 (75.4%)
Dry cough	102 (86.4%)
Hypoxaemia	67 (56.8%)

### Grocott–Gomori’s methenamine silver staining and DNA preparation of BALF samples

All operations of traditional Gomori’s methenamine silver (GMS) staining are strictly carried out in accordance with the literature [21]. Specifically, samples are placed in xylene for 15–20 min for deparaffinization and then hydrated by soaking them in 100% alcohol for 5 min, 95% alcohol for 5 min, 70% alcohol for 5 min and, finally, in distilled water for 5 min. Subsequently, the slides are immersed in a 5% chromic acid solution and oxidized at room temperature (20–25 °C) for 1 h to oxidize the polysaccharides in the fungal cell wall. After that, they are rinsed thoroughly with distilled water for 5–10 min. Next, the sections are treated with a 1% sodium bisulphite solution for 1 min to remove residual chromic acid and then rinsed with distilled water for 3–5 min. The slides are then immersed in the preheated Grocott’s methenamine silver nitrate solution (maintained at 60 °C), which contains 5% silver nitrate, 3% methenamine, 5% borax and distilled water, and stained in a water bath for 45 min whilst observing under a microscope until the fungal structures (such as *Pneumocystis* cysts) turn black. After staining, the slides are rinsed with distilled water for 3–5 min and then toned in a 0.1% gold chloride solution for 3 min to enhance contrast. Then, they are rinsed with distilled water for 3–5 min and treated with a 2% sodium thiosulphate solution for 2 min to remove background silver. Subsequently, they are counterstained with a light green solution (0.1% light green in 0.2% acetic acid) for 30 s to stain the background tissue. Finally, the slides are dehydrated by soaking them in 95% alcohol for 5 min and then in 100% alcohol for 5 min, cleared in xylene for 10–15 min and mounted with a resinous medium. The BALF sample was mixed evenly, and 1 ml was aspirated into a 1.5 ml sterile centrifuge tube and centrifuged at 8,000 r.p.m. for 5 min. The supernatant was discarded, leaving 50 µl of it over the pellet. Saponin (Tokyo Chemical Industry, S0019) was added to the vial at a final concentration of 5% (50 µl of 10% saponin), and the pellet was resuspended and mixed well by vibration and allowed to stand for 15 min at room temperature. This mixture was centrifuged at 12,000 r.p.m. for 2 min, and the supernatant was discarded. Two hundred microlitres of HL-SAN buffer (3 M NaCl and 50 mM MgCl2 in nuclease-free water) followed by 10 µl of HL-SAN DNase (25,000 units, Arcticzymes, 70910-202) were added to the pellet. The pellet was resuspended and mixed evenly by pipetting, digested at 37 °C for 10 min and heated at 80 °C for 2 min. Glass beads (100 mg) were added to this solution, and the vortex was vibrated at 2,000 r.p.m. for 8 min. Subsequently, DNA was extracted using the QIAamp DNA Mini Kit (Qiagen, Germany), which includes Buffer ATL (lysis buffer), Buffer AL (binding buffer) and ethanol. The protocol followed the manufacturer’s instructions, with modifications: 200 µl of HL-SAN buffer (3 M NaCl, 50 mM MgCl₂) and 10 µl of HL-SAN DNase were added to lyse human cells and degrade host DNA. DNA concentration was calibrated using the Qubit reagent (Q33230, Thermo Fisher Scientific, Invitrogen, Carlsbad, CA, USA). Saline solution (0.9% NaCl) was used as a blank control to monitor environmental contamination during DNA extraction, as nuclease-free water may lack ionic balance for comparative analysis.

### Real-time PCR

The extracted BALF DNA samples were analysed using a previously described real-time PJP PCR assay [22,23]. The assay is a qualitative, real-time PCR performed on the LightCycler instrument (Roche Molecular Diagnostics) using the LightCycler^®^ 480 Probes Master kit (Roche Diagnostics), containing FastStart Taq DNA Polymerase, dNTPs and MgCl₂, with primers and probes designed to target the cdc2 gene (GenBank accession: XM_017371254.1) using PrimerExpress v3.0 (Applied Biosystems). Dual probes enhance specificity by requiring dual fluorescence signals for positivity. The primers used were the following: forward (5′-AGGTAGGAGAAG GTAAGAAA-3′), reverse (5′-GCTGTGCTTGGAACCC-3′), probes (5′-GATCTTGAAAATGGCACAATAGTAG-fluoroscein-3′) and (5′-Red640-TTAAAAAAATCCGGCTAGAAGCAGAAG-phosphate-3′). All PCR reactions were performed using the LightCycler-FastStart DNA Master Hybridization Probes kit (Roche Diagnostics). Each reaction had a final concentration of 4 mmol l^−1^ MgCl_2_, 0.4 µmol l^−1^ Red-640–labelled probe, 0.2 µmol l^−1^ fluorescein-labelled probe and 0.5 µmol l^−1^ of each primer. The final volume of each reaction was 20 µl (15 µl PCR master mix and 5 µl DNA). The PCR was programmed as follows: 5 min at 95 °C, followed by 45 cycles (95 °C for 15 s, 55 °C for 20 s and 72 °C for 20 s), preceded by a melting programme starting at 37 °C with a 1 °C increment per 30 s interval until 85 °C. A CT (Cycle Threshold) value ≤40 was considered positive and validated using serial dilutions of *P. jirovecii* genomic DNA [limit of detection (LOD): 100 copies per millilitre].

### Nanopore-based metagenomic third-generation sequencing

The Mix (FRM) reaction solution [SQK-RPB004 Rapid PCR Barcoding Kit (Nanopore), Oxford Nanopore Technologies, Oxford, UK] was prepared in a 0.2 ml PCR tube, mixed evenly by mild stirring at 30 °C for 5 min and 80 °C for 1 min and cooled quickly on an ice box. After PCR, the product was transferred to a 1.5 ml centrifuge tube. An equal amount of 50 µl purified magnetic beads was added and mixed well. The mixture was allowed to stand for 5 min at room temperature and placed on a magnetic stand after instantaneous centrifugation. When the solution turned clear, the supernatant was discarded. Following two cycles of washing with 180 µl of 80% ethanol, the sample was instantaneously centrifuged at 3,000 r.p.m. for 1 min, placed on a magnetic stand for absorption of the residual ethanol and dried at room temperature by leaving the lid uncovered for 30 s. Subsequently, 10 µl of 10 mM Tris-HCl (pH=8.0, PHG0002, SAFC Biosciences, Lenexa, KS, USA) and 50 mM NaCl (S8210, Solarbio Life Sciences, Beijing, China) were added and mixed by gentle rotation. Magnetic beads were suspended in this solution and incubated for 2 min at room temperature and then placed on the magnetic stand. When the solution turned clear, the eluate was aspirated for later use. Qubit reagent was used to calibrate the concentration of each sample eluate. A loading mixture series of eluate (100–200 ng µl^−1^ in 10 µl final volume) was prepared, and the same absolute quantity of template supplemented with 1 µl Rapid Adapter solution [SQK-RPB004 Rapid PCR Barcoding Kit (Nanopore), Oxford Nanopore Technologies, Oxford, UK] was added, mixed gently and incubated at room temperature for 15 min. Subsequently, the loading mixture was sequenced. The sequencing data volume was 1GB.

### MinION library preparation and sequencing

The MinION library was prepared using three assay kits – (1) the Rapid Low-Input PCR Sequencing Kit (SQK-RLI001), (2) the Rapid Low-Input Barcoding Kit (SQK-RLB001) and (3) the Rapid PCR Barcoding Kit (SQK-RPB004) – according to the manufacturers’ instructions with minor alterations. Briefly, for single sample sequencing runs using the SQK-RLI001 kit, 10 ng of the MagNA Pure-extracted DNA was used for the tagmentation/fragmentation reaction, where DNA was incubated at 30 °C for 1 min and at 75 °C for 1 min. The PCR reaction was run as per the manufacturer’s instructions; however, the number of PCR cycles was increased to 20. For multiplexed runs, SQK-RLB001 and SQK-RPB004 kits were used. A 1.2×AMPure XP bead (Beckman Coulter, A63881) wash was introduced after the MagNA Pure DNA extraction and before library preparation for multiplexed runs, and DNA was eluted in 15 µl of nuclease-free water. Modifications used for the library preparation were as follows: (1) 10 ng of input DNA and 2.5 µl of FRM were used for the tagmentation/fragmentation reaction, and nuclease-free water was used to make up the volume to 10 µl, and (2) for the PCR reaction, 25 cycles were used, and the reaction volume was doubled. PCR was performed on samples using either the pilot or optimized methods. The pilot method used a 6 min extension time, whereas the optimized method used a reduced extension time of 4 min. For multiplexing, the PCR products were pooled together in equal concentrations, then subjected to a 0.6×AMPure XP bead wash and eluted in 14 µl of the buffer (10 µl of 50 mM NaCl, 10 mM Tris-HCl pH 8.0) recommended in the manufacturer’s manual. Sequencing was performed on the MinION platform using R9.4, R9.5 or R9.4.1 flow cells. The library (50–300 fmol) was loaded onto the flow cell according to the manufacturer’s instructions. ONT MinKNOW software (v.1.4–1.13.1) was used to collect raw sequencing data, and ONT Albacore (v.1.2.2–2.1.10) was used for local base-calling of the raw data after the sequencing runs were completed. The MinION was run for up to 48 h; WIMP/ARMA analysis was performed on the first six folders for pilot method samples and the first 2 h of data for all optimized method samples. If a species is detected in both the blank control and the test sample, but the number of reads for that species in the test sample exceeds three times that of the blank control, the species is retained. Otherwise, it is considered not to be a pathogenic micro-organism and is discarded.

### Statistical analysis

Statistical analysis was performed using SPSS v26. Sensitivity, specificity and accuracy were calculated with 95% CIs (Wilson score method).

## Results

### Detection of *P. jirovecii* using different methods

The metagenomics test detected *P. jirovecii* infections in 39 of the 118 patients with lung cancer with suspected PJP, giving a detection rate of 33.0% (39 out of 118). Meanwhile, the traditional GMS staining method and the real-time PCR method had detection rates of 4.2% (5 out of 118) and 30.5% (36 out of 118), respectively. Further, all 39 patients detected as infected with * P. jirovecii* and clinically diagnosed as having PJP recovered after targeted diagnostic treatment. Thus, using clinical diagnosis and post-treatment recovery as the basis for true detection (the base standard), we can say that positive rates for the metagenomics test, the GMS staining and the real-time PCR tests were 100% (39 out of 39), 12.8% (5 out of 39) and 92.3% (36 out of 39), respectively. Furthermore, the metagenomics test could detect all the positive cases, including those which went undetected by GMS staining and PCR tests. Additionally, the metagenomics test also picked up cases detected by GMS staining and PCR tests.

### Comparison of diagnostic efficacy between metagenomics, GMS staining and real-time PCR

Considering imaging, clinical presentation and treatment outcomes, 39 amongst 118 lung cancer patients with suspected PJP were clinically diagnosed with PJP, and 79 were not diagnosed with *P. jirovecii*. Using the clinical diagnostic treatment outcome as the basis for true detection (the base standard), the sensitivity of metagenomic testing was 100% (39 out of 39), which is higher than that of GMS staining [12.8% (5 out of 39) (5.1%–27.2%)] and real-time PCR [92.3% (36 out of 39) (79.0%–98.0%)] testing. Additionally, metagenomic testing demonstrated good specificity, equal to that shown by GMS staining and real-time PCR testing, which were both 100% (79 out of 79). The accuracy of metagenomics testing was 100% (118 out of 118), significantly higher than that of GMS staining [71.2% (84 out of 118) (62.4%–78.6%)], and slightly higher than that of real-time PCR testing [97.5% (115 out of 118) (92.5%–99.5 %)]. The positive predictive value and negative predictive value for metagenomic testing of *P. jirovecii* can both reach 100%; specific data are shown in Table 2. And comparative Ct values of real-time PCR and Nanopore output of the 39 metagenomic testing *P. jirovecii*-positive samples are shown in Table 3.

**Table 2.** Diagnostic performance of metagenomics vs. traditional methods

	Positive agreement rate (%)	Negative agreement rate (%)	Positive predictive value (%)	Negative predictive value (%)	Overall agreement rate (%)
Metagenomics	100	100	100	100	100
Gomori’s stain	12.8	100	100	80.7	71.2
Real-time PCR	92.3	100	100	96.3	97.5

**Table 3.** Composition, qPCR CT values and metagenomic sequence read outputs of metagenomics test positive of *P. jirovecii*

Specimen no.	qPCR result (CT)	Nanopore-based metagenomic reads	Abundance (%）
1	21	198,994	2.58
2	24.6	10,842	0.13
3	27.7	83,901	0.92
4	29.2	21,971	0.31
5	31.9	194	0.00
6	22.3	121,258	1.48
7	23.7	800,771	9.20
8	30.2	20,110	0.25
9	34.6	2,643	0.03
10	Undetermined	48	0.00
11	23.3	188,394	2.38
12	24.2	1,744,667	19.17
13	32.6	12,761	0.14
14	34.3	6,825	0.08
15	Undetermined	147	0.00
16	23.2	141,614	1.65
17	24.3	1,040,079	11.82
18	31.3	19,255	0.22
19	35	3,276	0.04
20	Undetermined	88	0.00
21	24.3	217,550	2.75
22	25.1	1,720,187	21.24
23	33.9	5,787	0.06
24	35.4	6,825	0.08
25	24.2	72,190	0.82
26	22.3	154,259	1.90
27	25.9	8,405	0.12
28	29	65,040	0.82
29	30.5	17,032	0.21
30	33.2	150	0.00
31	23.6	93,998	1.15
32	25	620,753	7.86
33	31.5	15,589	0.20
34	35.9	2,049	0.02
35	24.6	146,042	1.55
36	25.5	1,352,455	14.70
37	33.9	9,892	0.11
38	35.6	5,291	0.07
39	23.6	10,842	0.13

### Detection of other pathogens

**Fig. 1.**
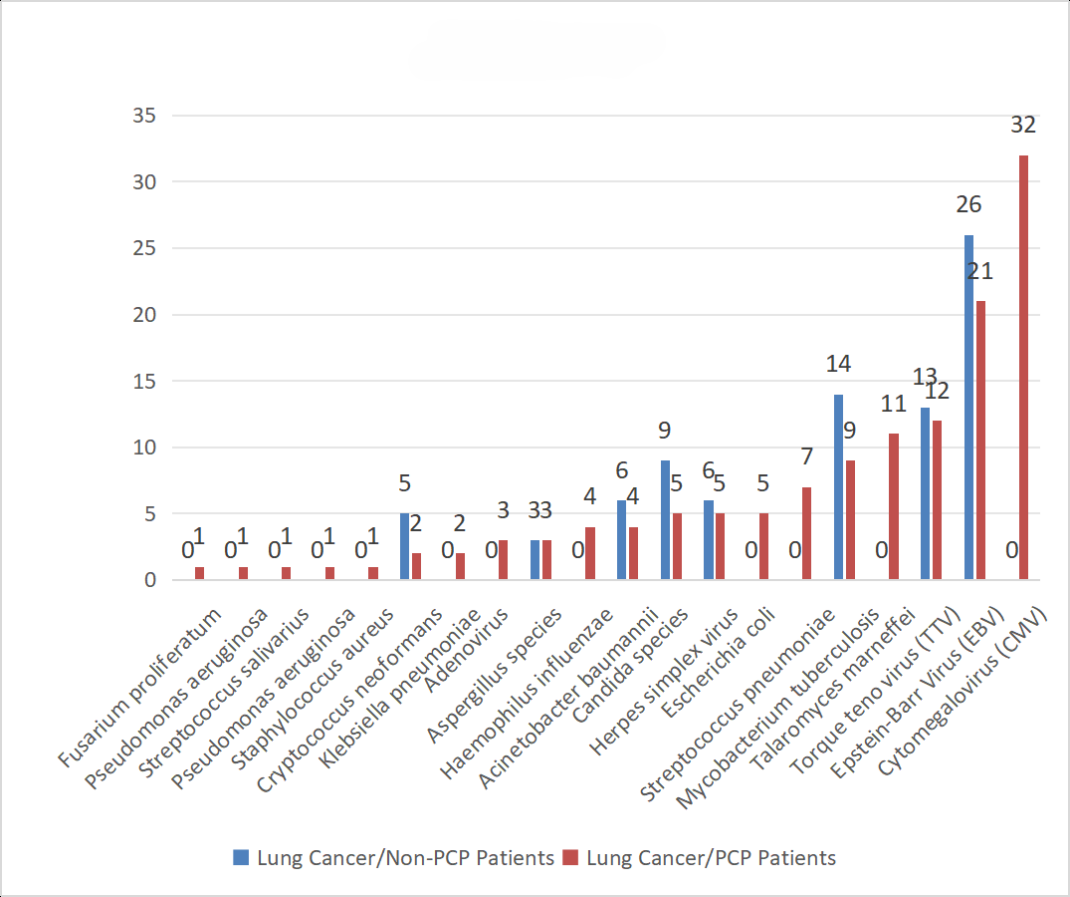
Co-infections detected by metagenomics in PJP vs. non-PJP patients. The Y-axis represents the number of detected pathogens in the study.

Co-infections were defined as ≥10 reads of a non-*P. jirovecii* pathogen after subtracting background (blank control reads ×3). Here, metagenomics successfully showed that many lung cancer/PJP patients were co-infected with other pathogens – such as *Cytomegalovirus* (CMV, *n*=32), Epstein–Barr virus (EBV, human herpesvirus 4, *n*=21), Torque teno virus (*n*=12) and *Talaromyces marneffei* (*n*=11). In addition, *Mycobacterium tuberculosis* was detected in nine lung cancer/PJP patients (Fig. 1). Amongst lung cancer/non-PJP patients, bacterial and viral co-infections (*n*=24) were the most common, followed by solely bacterial infections (*n*=20) and solely viral infections (*n*=17). Co-infections with fungi and viruses (*n*=6) were less frequent in lung cancer/non-PJP patients compared to those in lung cancer/PJP patients (*n*=12). Further, whilst triple infections with viruses, bacteria and fungi were lower in lung cancer/non-PJP patients (*n*=6), they were higher in lung cancer/PJP patients (*n*=24). We found that the 79 lung cancer/non-PJP patients were infected by fewer types of pathogens, which were mainly *M. tuberculosis* (*n*=14 cases) and EBV (*n*=26), along with other common fungi and viruses such as *Aspergillus* (*n*=3 cases), *Candida* (*n*=9) and herpesvirus (*n*=6).

## Discussion

*P. jirovecii* is an opportunistic pathogenic fungus that primarily causes latent infections in the lungs. If not treated promptly, the *P. jirovecii* infection can rapidly progress to acute respiratory distress syndrome, which is a major cause of death in cancer patients, especially in those with lung cancer [24]. Thus, timely diagnosis and precise symptomatic treatment of mixed infections of *P. jirovecii* in patients with lung cancer are crucial for lowering mortality rates.

*P. jirovecii* is difficult to culture *in vitro*. Thus, pathological diagnosis relies on laboratory-based microscopic identification of the pathogen in stained patient specimens. Traditional smear staining techniques include Giemsa staining, May–Grunwald–Giemsa staining, immunofluorescence staining and methenamine silver (Grocott) staining. Giemsa staining renders intracystic bodies purple-red but does not stain the cyst wall, causing poor contrast and potential underdetection. May–Grunwald–Giemsa staining poorly differentiates *P. jirovecii* from other fungi when the sample contains few scattered cysts. Immunofluorescence microscopy using fluorescent-labelled monoclonal antibodies against cysts and trophozoites provides greater accuracy when testing deep respiratory samples like BALF. It has been increasingly adopted due to its high sensitivity, convenience and speed. However, the immunofluorescence microscopy method is expensive and requires specialized microscopes, which limits its routine use in many hospitals. On the other hand, methenamine silver staining shows good diagnostic yield, revealing the characteristic ‘parentheses-shaped’ bodies (also called bracket-like structures) on the cyst wall that are unique to *P. jirovecii* and not present in other fungi, thereby minimizing the risk of underdetection. However, the positivity rate of GMS staining is influenced by factors such as staining methodology, quality of the specimen and organism load, and it is substantially subjective and requires technical skill [25].

Reported sensitivities for various staining methods across different samples are as follows: induced sputum, 50%–90%; BALF, 90%–99%; transbronchial biopsy, 95%–100%; and lung biopsy tissue, 95%–100% [26]. Spontaneously expectorated sputum samples are often unsuitable because they mostly contain saliva and oral secretions which make microscopic identification of * P. jirovecii* difficult. Hence, they are generally not used for *P. jirovecii* detection.

The current adjunctive diagnostic methods for *P. jirovecii* include the serum BDG test (G test) and molecular biological assays. Significantly elevated serum lactate dehydrogenase levels can also serve as a supportive diagnostic marker for PJP [27]. BDG is ubiquitous in fungal cell walls and can be markedly elevated in invasive fungal diseases, rendering it a nonspecific diagnostic marker unable to distinguish between fungal species. Thus, the G test result can be positive for various fungi belonging to the genera *Candida*, *Aspergillus*, *Fusarium*, *Scedosporium*, *Acremonium* and *Pneumocystis*, including other dematiaceous fungi [28]. PCR testing compares favourably with methenamine silver staining and fluorescence staining in terms of sensitivity and specificity, particularly when used on non-invasive samples. It is being increasingly used for identifying *Pneumocystis* genus members. Nonetheless, it has lower specificity than methenamine silver staining and may produce false positives. PCR detection of *P. jirovecii* (without corresponding microscopic evidence) could indicate colonization rather than infection, as it cannot differentiate between the two states. Hence, PCR testing is not a routine method for diagnosing *P. jirovecii* infections [26].

This study substantiated the advantages of metagenomics third-generation sequencing in diagnosing PJP in patients with lung cancer, demonstrating significantly higher positivity rates compared to traditional methenamine silver staining and real-time PCR; the metagenomic test had excellent sensitivity (100%) and specificity (100%). Actually, we have assessed the LOD for the metagenomics method in detecting *P. jirovecii*, and the LOD is 10 c.f.u. ml^−1^. Moreover, metagenomics detected concurrent infections with other pathogens in lung cancer patients with or without PJP, allowing for targeted treatments. Patients with lung cancer are prone to unexplained infections due to their immunocompromised state. Metagenomics effectively detected concurrent pathogens in this study, including CMV, EBV and *T. marneffei* (as shown in Fig. 1), which are challenging to identify using traditional culture-based methods. These findings align with the pathogens explicitly reported in the Results section. Therefore, metagenomics is more efficient than traditional methods in identifying infections in lung cancer patients with initial empirical antimicrobial treatment failures. It enables prompt adjustment of treatment strategies by accurately identifying microbes. Here, all 39 patients with lung cancer and PJP responded well to *P. jirovecii*-specific treatment. In this study, metagenomics identified *Aspergillus* species in three non-PJP patients, enabling targeted antifungal therapy. This demonstrates its clinical utility in guiding treatment for co-infections. Thus, metagenomics can also be applied to dovetail treatments in filamentous fungal infection cases with initial empirical treatment failure. Metagenomics detected *M. tuberculosis* in 14 lung cancer/non-PJP patients, highlighting its role in diagnosing tuberculosis co-infections, which were not the primary focus of this study but are critical for comprehensive patient management. Thus, it prevents underdiagnosis, which holds significant scientific implications in diagnosis and treatment. Metagenomics results combined with clinical data allowed for rapid correction and enhancement of initial empirical antimicrobial regimens – treating both patients with lung cancer with and without PJP infections – resulting in targeted and precise anti-infection therapies. This approach reduced costs and ultimately led to better clinical outcomes.

Notably, the 100% sensitivity and specificity (estimated using clinical diagnostic treatment outcome as the basis for true detection) exhibited by metagenomics, here, might be attributed to the relatively small sample size. An expanded sample size could provide more accurate results. From a clinical perspective, metagenomics offers the advantage of accurately analysing the complete microbial profile in patient samples. This is highly valuable for studying the aetiology of infectious diseases, particularly in detecting and identifying pathogens in unknown infectious diseases where traditional microscopy, culture-based methods and PCR tests (by being limited to known microbial gene sequences) give non-optimal results. The nanopore-based metagenomic approach described in the Methods section demonstrated high-throughput sequencing capabilities, enabling simultaneous detection of *P. jirovecii* and co-infecting pathogens within ≤6 h. Here, metagenomics not only identified the target pathogen but also detected co-infections with other pathogens (with CMV co-infections being the most common). Concurrent infections with bacteria, viruses and fungi were also frequently observed, guiding effective antimicrobial therapy.

In this study, since all the cases collected were lung cancer patients and no additional healthy individuals (who might have *P. jirovecii* colonization) were enrolled as controls, it was not possible to determine a threshold between colonization and infection based on the number of metagenomic sequencing reads. This is also a limitation of the study, as it cannot serve as a standalone diagnostic method to differentiate colonization from infection and requires the integration of other clinical examination results for comprehensive assessment.

In summary, metagenomics offers distinct advantages over traditional methods for diagnosing *P. jirovecii* infections in patients with lung cancer, with faster turnaround times, high sensitivity and specificity. Its early application assists in the rapid and accurate diagnosis of PJP. By detecting *P. jirovecii* infection, it directs the clinicians to initiate anti-*P. jirovecii* treatment in suspected PJP patients. Thus, it supports evidence-based drug use. Overall, metagenomics demonstrates significant clinical value in detecting concurrent infections with *P. jirovecii* and other pathogens in patients with lung cancer.

## Abbreviations

BALF: bronchoalveolar lavage fluid
BDG: 1,3-*β*-d-glucan
CMV: *Cytomegalovirus*
CT: cycle threshold
EBV: Epstein–Barr virus
GMS: Gomori’s methenamine silver
LOD: limit of detection
PJP: *Pneumocystis jirovecii* pneumonia
